# Supplementary material for: Effect of an Educational Intervention on Pupil’s Knowledge, Attitudes, Perceptions, and Behavior on Air Pollution in Public Schools in Pristina
Source: Eur J Investig Health Psychol Educ. 2025 May 2;15(5):69. doi: 10.3390/ejihpe15050069 (PMC12110580; doi:10.3390/ejihpe15050069)
Supplement: Supplementary file 1 [file ejihpe-15-00069-s001.zip › ejihpe-3442801-supplementary.pdf]

Rasch model test results on knowledge from the pretest (*n* = 2560 participants)

Likelihood Ratio test:

```
Andersen LR-test:
LR-value: 162.347
Chi-square df: 12
p-value:  0
```

Likelihood Ratio test including complete summary:

```
Andersen LR-test:
LR-value: 162.347
Chi-square df: 12
p-value:  0


Subject Subgroup: Raw Scores <= Median:
Log-likelihood:  -9156.627

Beta Parameters:
         beta Know_source_pollution beta Natural_source_poll
Estimate                -0.58223054             -1.12443658
Std.Err.                 0.05778988              0.06897542
         beta Small.partciles_Lung beta Greatest.contribution
Estimate               -0.91371717               -0.69908984
Std.Err.                0.06406822                0.05980899
         beta PM.2.5_source beta Know_Ozone beta Know_vul_groups
Estimate         0.39985666      -1.1086167           0.85627412
Std.Err.         0.04819678       0.0685809           0.04777196
         beta Know_same_vulner beta Know_prem_death beta Know_speed_cars
Estimate           -0.55341244           0.71832629          1.07347756
Std.Err.            0.05731878           0.04765346          0.04837703
         beta Know_staying.inside beta Know_air.quality_info
Estimate               0.58994163               -0.32940353
Std.Err.               0.04773311                0.05408882
         beta Know_disease
Estimate        1.67303059
Std.Err.        0.05265011


Subject Subgroup: Raw Scores > Median:
Log-likelihood:  -4755.036

Beta Parameters:
         beta Know_source_pollution beta Natural_source_poll
Estimate                -1.01961030             -0.95514952
Std.Err.                 0.06746385              0.06700142
         beta Small.partciles_Lung beta Greatest.contribution
Estimate               -0.75798099               -0.61766736
Std.Err.                0.06603192                0.06574575
         beta PM.2.5_source beta Know_Ozone beta Know_vul_groups
Estimate         0.76857415     -1.89495498           0.74719539
Std.Err.         0.08188776      0.08089348           0.08136012
         beta Know_same_vulner beta Know_prem_death beta Know_speed_cars
Estimate           -1.07101769           0.77576809          1.30596995
Std.Err.            0.06787931           0.08206748          0.09845138
         beta Know_staying.inside beta Know_air.quality_info
Estimate               0.54805186               -0.43863720
Std.Err.               0.07689362                0.06587334
         beta Know_disease
Estimate         2.6094586
Std.Err.         0.1720249
```

Graphical tests (including 95% confidence intervals) and checks for DIF:

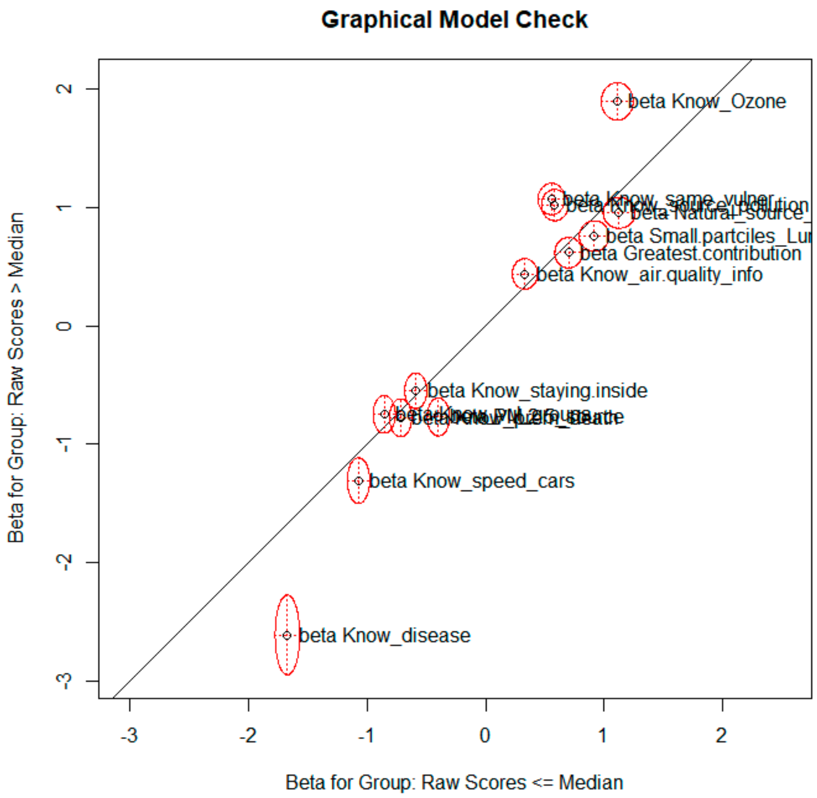

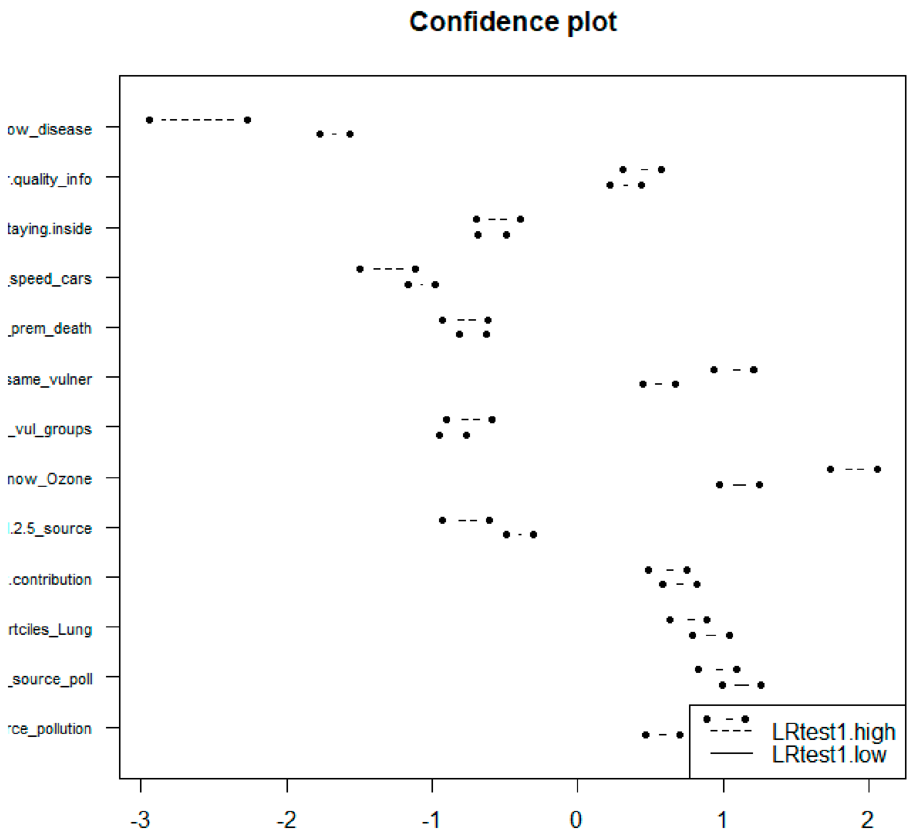

Wald test

```
Wald test on item level (z-values):

                              z-statistic p-value
beta Know_source_pollution          4.924   0.000
beta Natural_source_poll           -1.760   0.078
beta Small.partciles_Lung          -1.693   0.091
beta Greatest.contribution         -0.916   0.360
beta PM.2.5_source                 -3.880   0.000
beta Know_Ozone                     7.415   0.000
beta Know_vul_groups                1.156   0.248
beta Know_same_vulner               5.826   0.000
beta Know_prem_death               -0.605   0.545
beta Know_speed_cars               -2.119   0.034
beta Know_staying.inside            0.463   0.643
beta Know_air.quality_info          1.282   0.200
beta Know_disease                  -5.205   0.000
```

Rasch model: Item difficulty and easiness parameters with 95% CI:

```
Conditional log-likelihood: -13992.84
Number of iterations: 18
Number of parameters: 12

Item (Category) Difficulty Parameters (eta): with 0.95 CI:
                      Estimate Std. Error lower CI upper CI
Natural_source_poll      0.996      0.047    0.904    1.088
Small.partciles_Lung     0.798      0.045    0.710    0.886
Greatest.contribution    0.624      0.043    0.539    0.709
PM.2.5_source           -0.529      0.040   -0.608   -0.450
Know_Ozone               1.458      0.053    1.353    1.562
Know_vul_groups         -0.858      0.041   -0.939   -0.778
Know_same_vulner         0.753      0.044    0.666    0.840
Know_prem_death         -0.762      0.041   -0.842   -0.682
Know_speed_cars         -1.148      0.043   -1.232   -1.064
Know_staying.inside     -0.608      0.040   -0.687   -0.529
Know_air.quality_info    0.340      0.042    0.258    0.421
Know_disease            -1.809      0.049   -1.905   -1.713

Item Easiness Parameters (beta) with 0.95 CI:
                           Estimate Std. Error lower CI upper CI
beta Know_source_pollution   -0.746      0.044   -0.833   -0.659
beta Natural_source_poll     -0.996      0.047   -1.088   -0.904
beta Small.partciles_Lung    -0.798      0.045   -0.886   -0.710
beta Greatest.contribution   -0.624      0.043   -0.709   -0.539
beta PM.2.5_source            0.529      0.040    0.450    0.608
beta Know_Ozone              -1.458      0.053   -1.562   -1.353
beta Know_vul_groups          0.858      0.041    0.778    0.939
beta Know_same_vulner        -0.753      0.044   -0.840   -0.666
beta Know_prem_death          0.762      0.041    0.682    0.842
beta Know_speed_cars          1.148      0.043    1.064    1.232
beta Know_staying.inside      0.608      0.040    0.529    0.687
beta Know_air.quality_info   -0.340      0.042   -0.421   -0.258
beta Know_disease             1.809      0.049    1.713    1.905
```

Person parameters and person separation reliability:

```
Person Parameters:

 Raw Score   Estimate Std.Error
         0 -3.7890321        NA
         1 -2.8654949 1.0766276
         2 -2.0102641 0.8156062
         3 -1.4357328 0.7117170
         4 -0.9699477 0.6582822
         5 -0.5573820 0.6292532
         6 -0.1714370 0.6153263
         7  0.2047426 0.6132500
         8  0.5856265 0.6232382
         9  0.9882646 0.6489217
        10  1.4395452 0.6999182
        11  1.9952106 0.8026830
        12  2.8272476 1.0649426

> SepRel(pers)

Separation Reliability: 0.467
```
